# Investigations of organ and effective doses of abdominal cone-beam computed tomography during transarterial chemoembolization using Monte Carlo simulation

**DOI:** 10.1186/s12880-018-0247-7

**Published:** 2018-02-05

**Authors:** Yi-Shuan Hwang, Hui-Yu Tsai, Yu-Ying Lin, Kar-Wai Lui

**Affiliations:** 1 Department of Medical Imaging and Intervention, Chang Gung Memorial Hospital at Linkou, 5 Fushing Street, Kweishan, Taoyuan, 333 Taiwan, Republic of China; 2 Department of Medical Imaging and Radiological Sciences, College of Medicine, Chang Gung University, Taoyuan, Taiwan; 3 Institute of Nuclear Engineering and Science, National Tsing Hua University, Hsinchu, 300 Taiwan

**Keywords:** Cone-beam computed tomography, Effective dose, Organ dose, Conversion factor, Dose–area product

## Abstract

**Background:**

To investigate the organ dose, effective dose (ED), conversion factor, and the C-arm rotation angle effects on dose variations of abdominal C-arm cone-beam computed tomography (CBCT) during transarterial chemoembolization (TACE).

**Methods:**

The organ doses and EDs for abdominal C-arm CBCT were retrospectively calculated according to a Monte Carlo technique for 80 patients. Dose variations from projections, ED to dose–area product (DAP) ratios, and effects of body mass index (BMI) on the ED and ED to DAP ratios were also analyzed.

**Results:**

The kidney received the highest dose (14.6 ± 1.2 mSv). Organ dose deviations among C-arm rotation angles was highest for stomach (CV = 0.71). The mean ED of the the CBCT run during TACE was 3.5 ± 0.5 mSv, and decreased with increased BMI (R^2^ = 0.45, *p* < 0.001). The mean ED to DAP ratio was 0.27 ± 0.04 mSv·Gy^− 1^·cm^− 2^ and tended to decrease with increased BMI (R^2^ = 0.55, *p* < 0.001). The mean ED to DAP ratios were 0.29 ± 0.02, 0.26 ± 0.02, and 0.23 ± 0.03 mSv·Gy^− 1^·cm^− 2^ for patients with BMI < 25 kg/m^2^, 25–30 kg/m^2^, and ≥30 kg/m^2^, respectively.

**Conclusions:**

Suitable conversion factors for C-arm CBCT facilitate the use of DAPs for estimating the ED. The patient dose can be varied by adjusting the CBCT rotation angle setting, and dose reduction strategies can be further manipulated.

## Background

C-arm cone-beam computed tomography (CBCT), performed using an angiographic system that rotates a C-arm-mounted, flat-panel detector around the patient, is an imaging technique capable of yielding three-dimensional (3D) volumetric images. Although CBCT is useful for providing additional information on anatomical relationships, detecting tumors, determining the feeding arteries of tumors, and identifying the distribution of the contrast agent injected through the catheter [1], the additional radiation dose resulting from extra 3D imaging acquisitions during interventional procedures is difficult to evaluate in a patient [2].

The effective dose (ED) is considered the most appropriate quantity for estimating the stochastic risk of exposure to ionizing radiation. However, the complexity of dose calculations for C-arm CBCT complicates performing total ED estimations for patients who have undergone interventions, including fluoroscopic procedures and C-arm CBCT runs. Some studies have investigated the patient dose for abdominal CBCT procedures based on Monte Carlo simulations or Thermoluminescent Dosimeter (TLD) measurements [2–6]. To more rapidly estimate the ED, suitable conversion factors should be applied to the dose–area product (DAP) values [7]. Suzuki et al. surveyed three types of angiographic systems from three manufacturers and used three sizes of human-shaped phantoms with Monte Carlo simulations and TLD measurements to assess the doses and effects of the phantom size on the EDs for abdominal C-arm CBCT procedures [2, 5]. Their benchmark studies provided an important reference for abdominal CBCT dose investigations, and demonstrated that conversion factors are protocol specific and may differ among angiographic systems [2, 5].

This study estimated the organ dose, ED, and conversion factors for abdominal C-arm CBCT during transarterial chemoembolization (TACE) by using Monte Carlo simulations for the angiographic systems not included in previous studies. The C-arm rotation angle effects on the organ dose based on the simulations were also investigated. Additionally, the relationship between the ED and patient body mass index (BMI) was investigated, and conversion factors that convert the DAP to ED based on the BMI categories for C-arm CBCT acquisitions in TACE were proposed for more detailed ED estimations.

## Methods

### Patients

This retrospective study was conducted with approval from institutional review board, and patient informed consent was waived. Eighty patients with hepatocellular carcinoma (HCC; 56 men and 24 women; average age, 65 years) scheduled for TACE between June 2015 and January 2016 were included. None of these patients were optimal candidates for surgery or local treatments, such as radiofrequency ablation.

Patient BMI was calculated from the height and weight listed in the medical records. To further investigate the effects of patient BMI on the dose, the BMI was divided into three categories according to the World Health Organization and National Institutes of Health classification schemes [8]: < 25 kg/m^2^ (normal), 25–30 kg/m^2^ (overweight), and ≥ 30 kg/m^2^ (obese). The mean patient BMI was 25.3 ± 4.0 kg/m^2^, and the patient characteristics are summarized in Table 1.

**Table 1:** Patient characteristics and mean DAPs, EDs, and ED to DAP ratios

	Patient number (Male / Female)	Age	Height (cm)	Weight (kg)	DAP (Gy·cm^2^)	ED (mSv)	ED to DAP ratio (mSv·Gy^−1^·cm^− 2^)
All patients	80 (56/24)	65 ± 12 (36–87)	162 ± 8 (146–183)	67 ± 13 (48–120)	12.9 ± 0.8 (11.8–17.1)	3.5 ± 0.5 (2.1–4.5)	0.27 ± 0.04 (0.17–0.35)
BMI < 25 kg/m^2^	45 (30/15)	68 ± 11 (37–87)	161 ± 7 (148–176)	59 ± 6 (48–76)	12.9 ± 0.6 (12.3–15.4)	3.8 ± 0.3 (3.0–4.5)	0.29 ± 0.02 (0.24–0.35)
BMI 25–30 kg/m^2^	22 (18/4)	62 ± 12 (40–83)	163 ± 7 (146–173)	72 ± 7 (58–86)	12.8 ± 0.6 (11.8–14.8)	3.3 ± 0.4 (2.7–4.2)	0.26 ± 0.02 (0.22–0.30)
BMI ≥ 30 kg/m^2^	13 (8/5)	60 ± 13 (36–83)	163 ± 8 (148–183)	85 ± 13 (68–120)	13.2 ± 1.4 (11.9–17.1)	3.0 ± 0.4 (2.1–3.9)	0.23 ± 0.03 (0.17–0.28)

Data are mean ± SD (range)

### Angiographic system and C-arm CBCT application

In this study, BRANSIST safireVC17 (Shimadzu Corporation, Japan) equipped with a 17-in. direct-conversion flat-panel detector was used as the angiographic system. The system is available for C-arm CBCT applications without automatic exposure control (AEC) adjustments, and the exposure parameters, such as the tube voltage (kV) and current (mA), are kept constant during the C-arm rotation. All technical parameters of the C-arm CBCT application in patients with HCC during TACE are summarized in Table 2. CBCT images were acquired from right anterior oblique (RAO) 120° to left posterior oblique (LAO) 95°, with a total acquisition range of 215°.The DAP values were recorded by technologists and used as the dose index for the dose calculations for each patient.

**Table 2:** Clinical settings of abdominal CBCT acquisitions for patients undergoing TACE

	Configuration values and acquisition parameters
Acquisition mode	CB-CTAP
Acquisition rate (fps)	30
Radiation time (sec)	12
Rotation speed	20°/sec
Number of frames	Approximate 315 frames
Acquisition range	215**°**
Focus-to-image distance (FID) (cm)	120
Field of view size (inch)	17
Focus	Large focus
Total filtration (mm)	11.2-mm aluminum equivalent
kV	100
mA	360
Pulse width (msec)	5.6

### Organ dose, ED, and conversion factor analysis

Dose evaluations were performed using PCXMC (PCXMC 2.0; Radiation and Nuclear Safety Authority, Helsinki, Finland) according to a Monte Carlo technique. The anatomical data were determined using the hermaphrodite phantom models, and further modified the simulated phantom sizes based on the adult phantom model by adjusting the weights and heights to mimic those of the patients. According to the adjusted weight and height, the program modified the phantom sizes and shapes by using calculated vertical and horizontal scaling factors [9].

In the simulation, the doses were calculated for 29 organs in 44 projections at 5°-intervals from RAO 120° to LAO 95°. To calculate the organ doses for each projection, the projection data including tube voltage (kVp), target angle, filtration, projection angle, X-ray field entrance position, focus to the patient skin distance (FSD), and the field size, were served as input parameters for the program.

The DAP is the input-dose quantity supplied for dose calculations, and organ dose and ED for each projection could thus be calculated subsequently. Notably, the ED was estimated using the tissue weighting factors defined in the International Commission on Radiological Protection report No. 103 [10]. The reported organ dose and ED for each patient was calculated by summing the doses in all 44 projections.

To further estimate the the angular dependence of the organ dose, the mean and standard deviation (SD) from the 44 projections for each organ dose as well as the coefficient of variation (CV) defined as the SD normalized to the mean were calculated. The ED to DAP ratio, defined as the ED normalized to the DAP value, was also calculated for the C-arm CBCT procedure performed on each patient [2, 5, 11, 12]. Thus, conversion factors for the CBCT performed during TACE were estimated based on the ED to DAP ratios calculated from patient data. Finally, the effects of patient BMI on the ED and ED to DAP ratios were also investigated.

### Statistical analysis

SPSS software (version 18.0; SPSS, Chicago, Illinois) was used for statistical analysis. Correlations between patients’ BMI and EDs as well as EDs to DAP ratios of CBCT were performed using linear regression. Two-sided *p* values < 0.05 were considered significant. The magnitudes of the differences of the mean EDs as well as mean ED to DAP ratios between three BMI categories were analyzed by one-way analysis of variance (ANOVA). The differences were considered significant at *p* values < 0.05.

## Results

### Organ dose and ED calculations

**Fig. 1:**
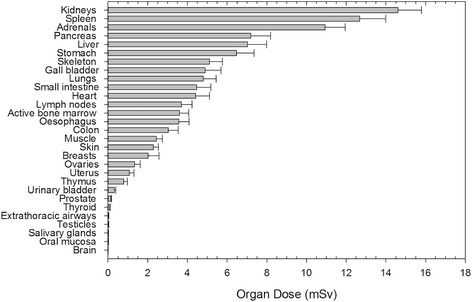
Mean organ dose calculated using PCXMC for the CBCT acquisitions for TACE. Error bars indicate SDs from 80 patients

The mean DAP of the CBCT run during TACE was 12.9 ± 0.8 Gy·cm^2^ (11.8–17.1 Gy·cm^2^; Table 1). Additionally, Fig. 1 presents the organ dose distributions. The following organs received the highest organ dose: kidney (14.6 ± 1.2 mSv), spleen (12.7 ± 1.3 mSv), adrenal gland (10.9 ± 1.0 mSv), pancreas (7.2 ± 1.0 mSv), and liver (7.0 ± 1.0 mSv). The mean ED per CBCT acquisition was 3.5 ± 0.5 mSv, and ranged from 2.1 to 4.5 mSv.

### Effects of patient BMI on ED

**Fig. 2:**
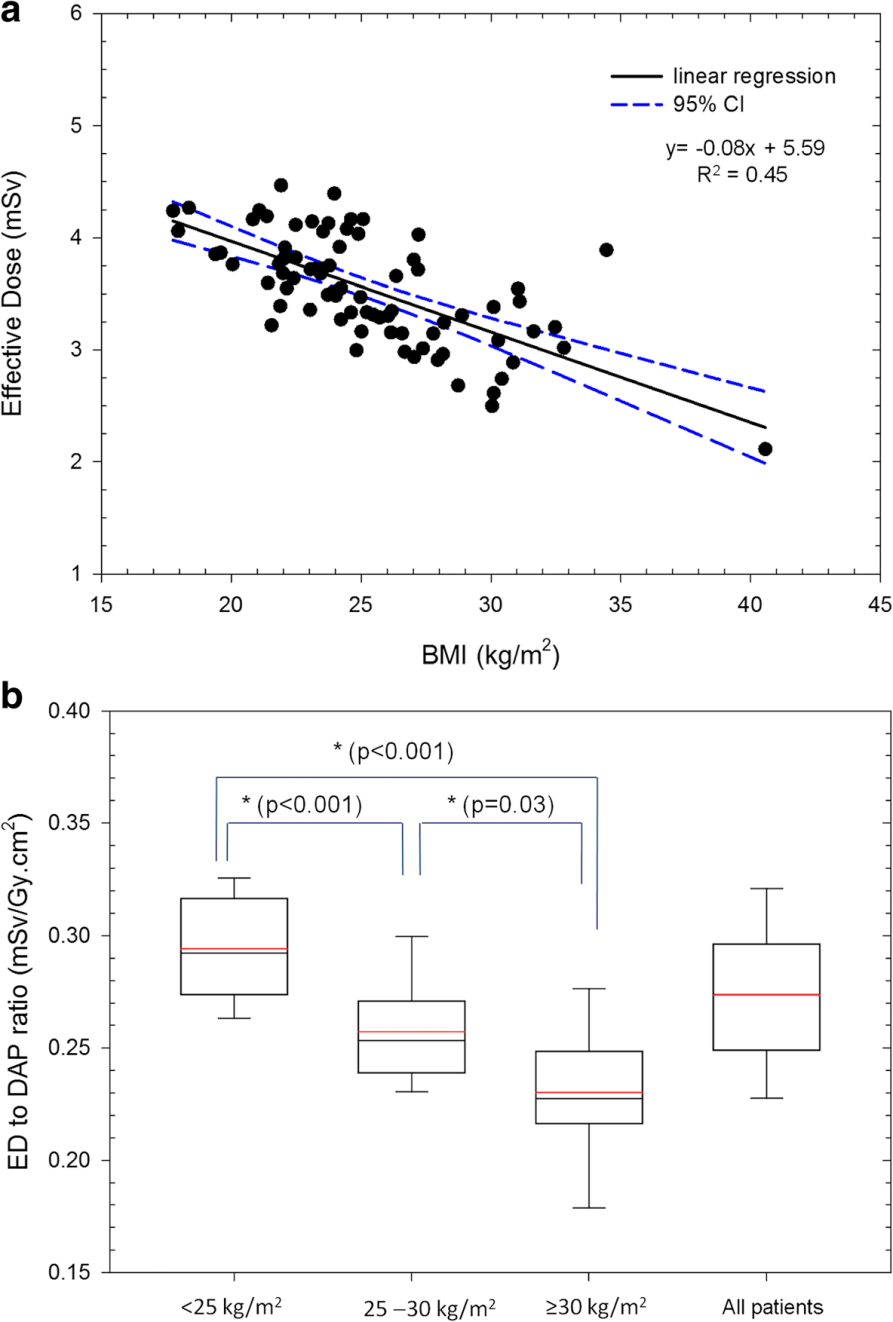
ED and ED to DAP ratio dependence on patient BMI. **a** An inverse linear relationship between the ED and BMI is demonstrated. Black solid line is the linear regression line between the ED and BMI, and the blue dashed lines display the range of 95% confidence interval (CI). **b** Box plots show the 25%–75% interquartile range of the ED to DAP ratio with patient BMI < 25 kg/m^2^, 25–30 kg/m^2^, ≥ 30 kg/m^2^, and with all the patients. Significant differences were observed in the ED to DAP ratio among the three BMI categories. Red and black lines indicate the mean and median values, respectively

Dependence of the ED on patient BMI during the CBCT acquisitions is demonstrated in Fig. 2a, the ED decreased slightly with increased patient BMI (R^2^ = 0.45, *p* < 0.001). A significant difference was observed in the ED among the three BMI categories (*p* < 0.05, one-way ANOVA). The mean ED values were 3.8 ± 0.3, 3.3 ± 0.4, and 3.0 ± 0.4 mSv for normal, overweight, and obese patients, respectively (Table 1).

### Conversion of DAP values to ED

In the clinical C-arm CBCT acquisitions for TACE investigated, the calculated mean ED to DAP ratio was 0.27 ± 0.04 mSv·Gy^− 1^·cm^− 2^ for the entire patient population (0.17–0.35 mSv·Gy^− 1^·cm^− 2^). However, the results also revealed that the ED to DAP ratio was highly dependent on patient BMI and it decreased linearly with increased patient BMI (R^2^ = 0.55, *p* < 0.001).

Furthermore, significant differences were observed in the ED to DAP ratios among the three BMI categories; specifically, the mean values were 0.29 ± 0.02, 0.26 ± 0.02, and 0.23 ± 0.03 mSv·Gy^− 1^·cm^− 2^ for normal, overweight, and obese patients, respectively (Fig. 2b; *p* < 0.05, one-way ANOVA). The mean ED to DAP ratios estimated from the entire patient population and from the BMI categories served as the conversion factors for the Shimadzu angiographic system, and the values are listed in Table 1.

### Effects of C-arm rotation angle on organ doses

**Fig. 3:**
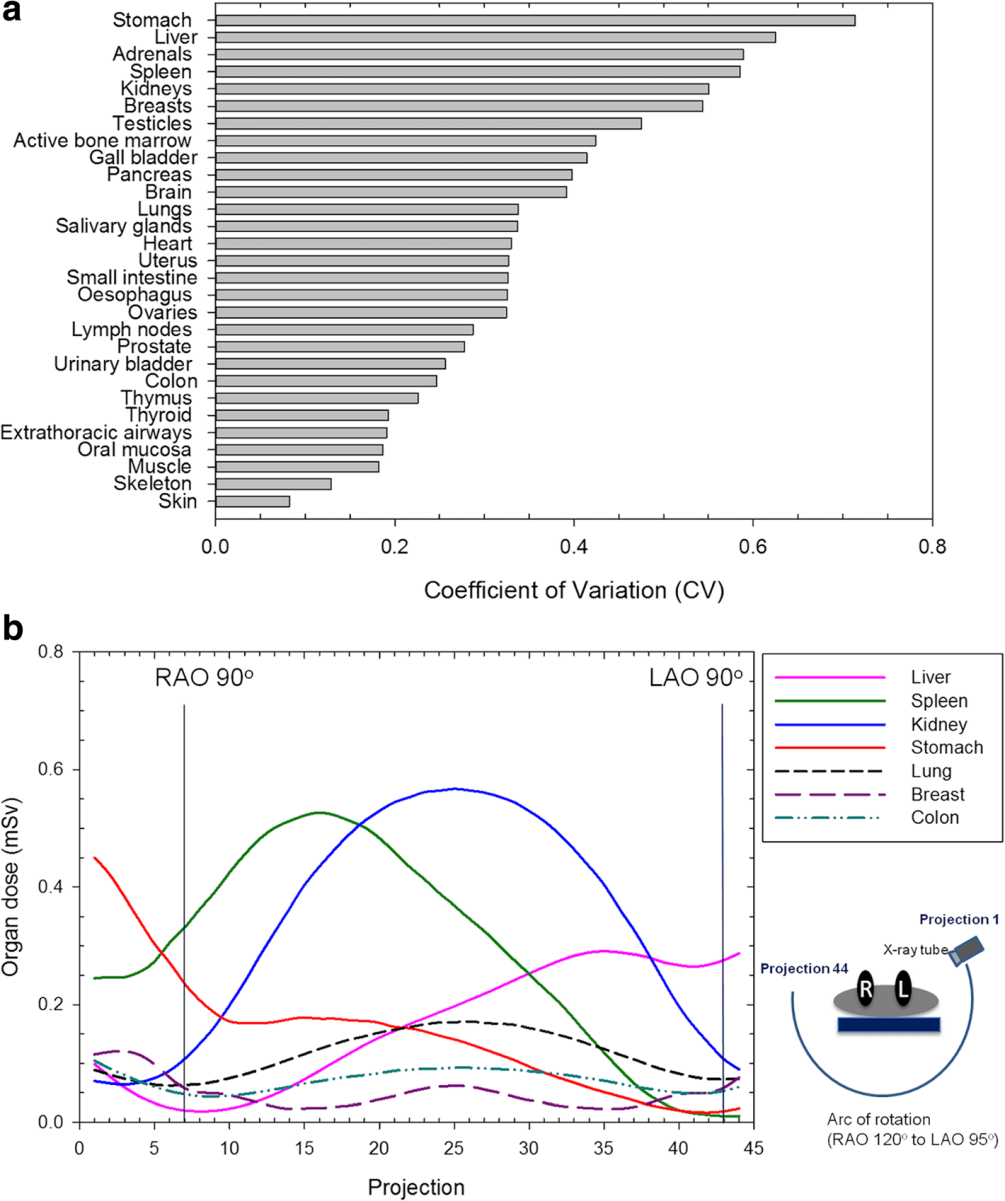
Dose variations and angular dependence of the organ dose during X-ray tube rotation. **a** Organ dose variations from different X-ray irradiated angle, represent as CVs of the dose from the 44 simulated projections for each organ. **b** The distribution of the demonstrated organ dose per projection for the CBCT procedure

The CVs of the organ dose calculated from the 44 projections demonstrated deviations among rotation angles are illustrated in Fig. 3a. The results revealed the highest variations for the stomach (CV = 0.71), followed by the liver (CV = 0.62), adrenal gland (CV = 0.59), spleen (CV = 0.59) and kidney (CV =0.55).

To further demonstrate the angular dependence of the dose absorbed by the organs during X-ray tube rotation, Fig. 3b illustrates the organ dose for organs with a higher absorbed dose or higher dose variations from the aforementioned 44 projections (e.g., kidney, spleen, liver, and stomach) and some radiosensitive organs (e.g., lung, breast, and colon) for each projection of the CBCT procedures during the entire tube rotation. An evaluation of the organ locations and projection angles revealed that the dose absorbed by the liver increased from approximately projection 8 to projection 35 (corresponding to RAO 85° to LAO 50°), and the dose distributions for both kidney and spleen demonstrated wide peaks during X-ray tube rotation through the posterior sides of the patients.

## Discussion

TACE is an angiographic procedure used to treat patients with hepatic tumors by injecting chemotherapeutic drugs into the selected hepatic artery [13]. To achieve more detailed patient dose investigations during these procedures, the ED obtained through fluoroscopy and CBCT imaging acquisitions should be individually evaluated. Multiple vendors have offered C-arm CBCT applications for angiographic systems, and the dose performance may vary because of the effects of varying designs on the beam quality as well as different protocol settings. To provide additional dose assessment information on abdominal CBCT, in addition to using popular angiographic units used in previous studies [2–6], organ dose and ED for C-arm CBCT acquisitions during hepatic TACE with the angiographic system without AEC capability when performing CBCT acquisitions by using Monte Carlo simulations were investigated.

Studies have investigated the doses for abdominal CBCT, as summarized in Table 3. The mean ED for the CBCT run was determined as 3.5 ± 0.5 mSv in this study. These values were slightly higher than the dose calculated according to the same Monte Carlo technique for the medium phantom with C-arm CBCT acquisitions with a GE INNOVA 4100 (3.1 mSv) but lower than the dose calculated for the large phantom with C-arm CBCT acquisitions using the same GE angiographic system (3.8 mSv) in the study by Suzuki [2].

**Table 3:** Previously reported EDs and ED to DAP ratios for abdominal CBCT procedures

Authors	Procedure	Scanner model	Rotation	Dose estimation method	Effective dose (ED) (mSv)	ED to DAP ratio (mSv·Gy^− 1^·cm^− 2^)
This study	Abdominal CBCT imaging: CB CTAP	Shimadzu BRANSIST safireVC17	215^o^	Using PCXMC based on individual patient data	3.5 ± 0.5 (2.1–4.5)	0.17–0.35
Suzuki et al. [2]	Abdominal 3D imaging	Philips Allura Xper FD20/10	207 ^o^	(1) Placing TLD in the human-shaped phantom (S) (2) Using PCXMC for three human-shaped phantom	(1) TLD: 1.6 (2) PCXMC: 1.9 (S), 2.5 (M), 3.1 (L)	0.37–0.45
		GE INNOVA 4100	194^o^		(1) TLD: 2.0 (2) PCXMC: 2.2 (S), 3.1 (M), 3.8 (L)	0.26–0.32
		Siemens AXIOM Artis dTA	200^o^		(1) TLD: 2.6 (2) PCXMC: 2.1 (S), 2.4 (M), 2.6 (L)	0.13–0.15
Braak et al. [3]	CBCT guidance (upper abdomen)	Philips XperCT Allura FD20	240^o^	Using PCXMC based on individual patient data	4.2 (95% CI 3.8–4.6)	N/A
Kwok et al. [4]	Abdominal CBCT imaging: DynaCT 8-s DR	Siemens Artis zeego	200^o^	Placing TLD in the human-shaped phantom	15	N/A
	Abdominal CBCT imaging: LCI CTHA Low 10 s	Toshiba Infinix VC-i	200^o^		25.4	N/A
Sailer et al. [6]	CT abdomen LD roll	Philips XperCT Allura FD20	180^o^	Using PCXMC based on individual patient data	4.3 (95% CI 3.9–4.8)	N/A

*TLD* Thermoluminescent Dosimeter, *N/A* not available, *S* small phantom size, *M* medium phantom size, *L* large phantom size

In this study, BMI scores were used to analyze the relationship between patient size and the ED. The results revealed that the ED during C-arm CBCT acquisitions decreased with increased patient size, which is consistent with the findings of Wielandts et al. [9, 14] but contrasted with those of Ector et al. [8] and Suzuki et al. [2, 5]. Suzuki et al. demonstrated that the DAPs and ED increased with increased phantom size in the abdominal CBCT procedures with all three angiographic systems they investigated [2]. Wielandts et al. calculated CBCT doses for the ablation of arrhythmias and reported that ED is inversely related to patient BMI; this tendency may be because of the very limited increase in the DAP with the BMI despite AEC execution [14]. In this study, because of the technical specifications of the investigated angiographic system, the exposure parameters were preset in the CBCT procedures, regardless of patient size variations, and AEC was not activated in the acquisitions. Thus, DAP values deviated only slightly among the patients in different BMI groups. When the same exposure is used for patients with a higher BMI, the X-ray beam is attenuated more before being absorbed by the organ; thus, the organ doses and EDs would be lower for patients with a higher BMI than for those with a lower BMI. The effects of patient size on the ED for CBCT in systems with fixed-exposure techniques would be similar to those in systems with limited AEC modulations.

The establishment of conversion factors provide an approach for ED estimations when the DAP is available during angiography; however, the conversion factors differ among the angiographic systems and are specific to the used imaging protocols. Suzuki et al. estimated the ED to DAP ratios for three phantom sizes by using three types of angiographic systems for 3D abdominal imaging procedures [2]. Notably, in their survey, patient height and weight affected the ED to DAP ratios slightly, whereas the ED to DAP ratios were 0.37–0.45, 0.26–0.32, and 0.13–0.15 mSv·Gy^− 1^·cm^− 2^ on the Philips Allura Xper FD20/10, GE INNOVA 4100, and Siemens AXIOM Artis dTA systems, respectively, and the conversion factors were estimated to be approximately 0.4, 0.3, and 0.15 mSv·Gy^− 1^·cm^− 2^ on these three systems. Thus, Suzuki et al. concluded that the ED for each patient can be easily estimated using a suitable conversion factor set for each angiographic system [2].

To more conveniently evaluate the EDs for patients, our methodology was based on Monte Carlo simulations, and patient data were collected as input for dose calculations. In this study, the mean ED to DAP ratio was 0.27 ± 0.04 mSv·Gy^− 1^·cm^− 2^ on a Shimadzu BRANSIST safireVC17 for all patients, and the ratios decreased with increased BMI. The trend was similar with the results of Suzuki et al., and studies have reported that the body volume percentage in the exposure field decreases with increasing phantom size, thus contributing to the effects [2, 5]. The mean ED to DAP ratio estimated from all patients can serve as a conversion factor for easier ED estimations when the DAP is available. However, because of the strong effects of patient BMI on the ED to DAP ratios, using conversion factors without considering patient size may result in the overestimation of ED for obese patients. Using conversion factors adapted to patient size in addition to the DAP can serve as a feedback mechanism for providing clinicians with more details on ED estimations when performing C-arm CBCT.

The variation in the relative position of the organs to the X-ray tube during the C-arm rotation explains the fluctuations in the projection-by-projection radiation dose. The dose was higher when the organ was irradiated by the incident beam. During the C-arm rotation, dorsal organs, such as kidneys, received a higher dose during the major portions of the projections because the C-arm rotated from the left to the right side of the patient and went through the posterior side during the acquisitions. Among the 44 projections, dose variation was the highest for the stomach, followed by the liver, possibly because the stomach and liver were irradiated directly in the start and end positions, respectively, of the C-arm rotation. The dose absorbed by the liver increased during the rotation because the liver was more directly irradiated with the incident beam aimed at the anterior right side of the patient. By contrast, the stomach absorbed higher doses in the initial projections because of the direct X-ray irradiation in the beginning of the rotation trajectory, and the dose decreased during the rotation.

When totaling the organ doses from all 44 projections, the simulated results revealed that total doses to the organs in the upper abdomen were higher than those to the other organs. This is because the liver was the target organ during C-arm CBCT acquisitions. Therefore, the radiation window was always positioned in the upper abdomen during the C-arm rotation. In this study, dorsal organs, such as the kidney and adrenal gland, received the highest total dose during C-arm CBCT acquisitions, which likely occurred because the C-arm was rotated through the posterior side of the patients during the acquisitions, and the mentioned organs were localized in the direct-irradiated FOV for major portions of the projections during the exposure procedure. Notably, this phenomenon is in strong concordance with the results of Suzuki et al. [2, 5].

ED is calculated as a weighted sum of organ doses; therefore, its value is mainly determined by the organs that are highly irradiated and those that have more crucial weighting factors [9]. Based on the finding that the organ dose would vary in all radiation projections, ED for patients can be varied by adjusting the C-arm CBCT rotational angle. The stomach is one of the most radiosensitive organs (tissue weight factor, 0.12) [10] in the irradiated FOV; therefore, decreasing stomach dose by adjusting the C-arm CBCT rotational angle may lead to largely decrease total ED. C-arm rotation angle, FOV locations as well as the C-arm rotating around the anterior or posterior sides of the patients markedly affected patient doses, and this indicated that dose reduction strategies can be further manipulated from C-arm rotation angle setting or X-ray irradiated field location.

Our study has some limitations. First, the applicability of the results may be restricted. A system without AEC for C-arm CBCT applications was used in this study, and the results may be applicable to similar system configurations but not to those with AECs. However, the methodology described herein can still be used as a reference for patient dose evaluations of C-arm CBCT acquisitions. To provide more feasible clinical applications, the conversion factors for C-arm CBCT acquisitions should be further evaluated for different protocols and other angiographic systems from diverse manufacturers. Second, the use of the BMI as a patient size indicator has limitations. For example, a muscular patient with a narrow waist and an overweight patient can have a similar BMI, and a patient with ascites may have a low BMI but increased abdominal girth, which would affect the organ dose as well as the ED; this may not be reflected in the simulations.

## Conclusions

We calculated the organ dose and ED according to a Monte Carlo technique for C-arm CBCT acquisitions during TACE by using a Shimadzu BRANSIST safireVC17 system. The ED to DAP ratios may differ with the protocols, systems, and patient sizes; however, overall, both ED and ED to DAP ratios decrease with increasing patient size. Suitable conversion factors for C-arm CBCT acquisitions facilitate the use of DAPs for estimating the ED during CBCT procedures and thus provide convenient patient dose estimations. The radiation dose absorbed by patients can be varied by adjusting the C-arm CBCT rotational angle settings, and dose reduction strategies can be further manipulated.

## Abbreviations

3D: Three-dimensional
AEC: Automatic exposure control
ANOVA: Analysis of variance
BMI: Body mass index
CBCT: Cone-beam computed tomography
CV: Coefficient of variation
DAP: Dose–area product
ED: Effective dose
FSD: Focus to the patient skin distance
HCC: Hepatocellular carcinoma
kV: Tube voltage
LAO: Left posterior oblique
RAO: Right anterior oblique
SD: Standard deviation
TACE: Transarterial chemoembolization
TLD: Thermoluminescent Dosimeter
